# Different clinical features of children and adults in regional outbreak of Delta COVID-19

**DOI:** 10.1186/s12879-022-07707-6

**Published:** 2022-09-08

**Authors:** Mei-Zhu Hong, Rongxian Qiu, Wei Chen, Hui Lin, Qing-Qing Xing, Xuan Dong, Jin-Shui Pan, Qin Li

**Affiliations:** 1 Department of Traditional Chinese Medicine, Mengchao Hepatobiliary Hospital of Fujian Medical University, No. 312, Xihong Road, Fuzhou, 350025 Fujian China; 2 Department of Infectious Diseases, Affiliated Hospital of Putian College, Putian, Fujian China; 3 Department of Hepatology, The First Affiliated Hospital of Fujian Medical University, No. 20, Chazhong Road, Fuzhou, 350005 Fujian China; 4 Hepatology Research Institute, Fujian Medical University, No. 20, Chazhong Road, Fujian 350005 Fuzhou, China

**Keywords:** COVID-19, Adult, Child, Clinical chemistry, Symptom assessment

## Abstract

**Background:**

This study compared clinical features of the Delta variant of coronavirus disease 2019 (COVID-19) in children and adults.

**Methods:**

Clinical data included 80 children and 132 adults with the Delta variant of COVID-19, hospitalized in the Affiliated Hospital of Putian College between September and October 2021. The data was analyzed retrospectively.

**Results:**

The proportion of mild patients in the children group (50%) was higher than that in the adults group (17.9%). Cough (25%, 20/80) and diarrhea (1.3%, 1/80) symptoms in children group were significantly less frequent. Compared with adults, there was no significant difference in the viral load of SARS-CoV-2 in samples collected by nasopharyngeal swabs. In children, lymphocyte count was higher [1.98 (0.25–4.25) vs 1.20 (0.29–4.27) ×10^9^/L], whereas the interleukin-6 level was lower [5.87 (1.50–61.40) vs 15.15 (1.79–166.30) pg/mL] than that in adults group. Additionally, the incidence of liver injury in children group was lower than that in adults group. There was no significant difference in the incidence of proteinuria (22/75 vs 45/112) between the two groups, but the serum creatinine level in children was lower [42.0 (28.0–73.0) vs 57.0 (32.0–94.0) µmol/L].

**Conclusion:**

Compared with adults, children with the Delta variant of COVID-19 have differences in symptoms, clinical classification, inflammatory indices, and liver/kidney function injury. Children’s illness is relatively mild. Clinicians should pay attention to their differences and use drugs accurately.

**Supplementary Information:**

The online version contains supplementary material available at 10.1186/s12879-022-07707-6.

## Introduction

Coronavirus disease 2019 (COVID-19), is a new acute respiratory infectious disease caused by the severe acute respiratory syndrome coronavirus 2 (SARS-CoV-2). The disease broke out at the end of 2019, with fever, dry cough, and fatigue as the main manifestations. In severe cases, it can rapidly progress to acute respiratory distress syndrome, multiple organ failure and even death, which poses a great threat to human health and life worldwide. In September 2021, COVID-19 broke out in Putian, Fujian province. Its pathogen was identified as the novel coronavirus Delta variant by virus gene sequencing. Most of studies focus on the clinical features of adults with COVID-19 while fewer studies report the clinical features of children [1–4]. However, children have intrinsic physiological properties which leads to significant difference from those of adults. The aim of this study was to analyze and compare the clinical characteristics of children and adults infected with the Delta variant of COVID-19, to improve the clinicians’ understanding of this disease and provide a reference for future clinical diagnosis and treatment.

## Patients and methods

### Patient details and setting

Between September and October 2021, 80 children (aged ≤ 14 years) and 132 adults (aged > 14 years) infected with the Delta variant of COVID-19 were hospitalized in the Affiliated Hospital of Putian University. The Institutional Review Board Mengchao Hepatobiliary Hospital of Fujian Medical University granted approval for this research. All methods were performed in accordance with the relevant guidelines and regulations.

### Diagnostic criteria

The diagnostic criteria and clinical classification criteria of confirmed cases with COVID-19 were in accordance with the national *Diagnosis and Treatment Protocol for COVID-19 Patients* (*Tentative 8th Edition*) [5]. Inclusion criteria was confirmed cases of COVID-19. Exclusion criteria was patients with serious mental disorders who cannot cooperate in the examination and treatment, patients with multisystem inflammatory syndrome. Liver injury usually manifests abnormal liver biochemistry with or without pathological changes. The abnormal liver biochemistry usually includes elevated ALT and/or AST, increased bilirubin, and decreased albumin. Elevated AST caused by myocardial or skeletal muscle injury should be excluded. Renal injury occurs as abnormal renal biochemistry with or without pathological changes. It is characterized by elevated creatinine and/or urea nitrogen, and/or pathological proteinuria. Proteinuria indicates by positive urine protein in the urine examination. However, strenuous exercise, tension, and other stress states can also lead to transient proteinuria and postural proteinuria.

### Research method

Data of children and adults infected with the Delta variant of COVID-19 were collected and analyzed retrospectively, including clinical manifestations, laboratory test results, and lung computed tomography (CT) examination.

### PCR testing instruments and reagents

Nasopharyngeal swabs were tested by fluorescence PCR, using the novel Coronavirus nucleic acid detection kit (Chongqing Zhengyuan Huiji Biotechnology Co., Ltd.) and a real-time fluorescence quantitative PCR instrument system (ABI QuantStudio 5). The viral load was expressed by the cycle threshold (CT) value. Interleukin-6 (IL-6) was detected using the IL-6 detection kit (Ji Dan Biotechnology Co., Ltd.) and a Getein 1600 fluorescence immunoquantitative analyzer.

### Statistical analysis

SPSS 25.0 software was used to analyze the data. The measurement data conforming to normal distribution was expressed as mean ± standard deviation. The *t*-test was used to compare the mean of the two groups for homogeneity of variance, and *t*-test was used for non-uniformity of variance. The measurement data that did not conform to normal distribution was described by median (lowest value, highest value), and was compared by nonparametric test. Frequencies were expressed by the number of cases and percentage in each category. Chi-square test was used to compare the counting data rate between the two groups, and the difference was statistically significant when a *P* value was ≤ 0.05.

## Results

### General information

Eighty children including 45 males and 35 females, aged between 3 and 13 years, with a median age of 9.0 years, were included in the study. There were 40 children with mild clinical classification (21 males and 19 females), and there were 40 patients with moderate type illness (24 males and 16 females) (Additional file 1: Table S1). There was no significant difference in the sex ratio between the two clinical types (*P* = 0.6525). Among children, one patient suffered from Glucose-6-Phosphate Dehydrogenase deficiency, one patient suffered from allergic rhinitis, and the rest had no obvious comorbidities. All the enrolled children have no previous renal diseases.

In the 132 adult patients (45 males and 87 females), aged between 16 and 86 years (median = 39.0) years. 24 patients presented with mild clinical classification (11 males and 13 females), and 108 presented with moderate infection (34 males and 74 females). No significant difference was observed between sexes in for mild and moderate infections (*P* = 0.18). Among the comorbidities reported by adult patients were diabetes mellitus (n = 8), hypertension (n = 9), renal insufficiency (n = 1), leukemia (n = 1), menstrual disorder (n = 1), chronic HBV infection (n = 1), allergic rhinitis and sinusitis (n = 3), and hysteria (n = 1).

The proportion of mild infection in children was 50% (40/80), significantly higher (*P* < 0.01) than that in adults of 18.2% (24/132; Table 2). The proportion of females in children was significantly lower at 43.8% (35/80) than in adults at 65.9% (87/132) (*P* = 0.001).

### Comparison of clinical symptoms

Differences in the clinical symptoms in children and adults are shown in Table 1. No significant differences were observed between the adult and children groups for fever (54/80 vs 73/132, *P* = 0.061) nor for pharyngeal discomfort (3/80 vs 15/132, *P* = 0.058). However, significant differences were noted between the two groups for coughing (20/80 vs 63/132, *P* = 0.001) and diarrhea (1/80 vs 19/132, *P* = 0.002), both of which were lower in children (Table 1).

**Table 1:** Comparison of clinical symptoms between children and adults

Symptom	Number of children (80 cases in total)	Number of adults (132 cases in total)	P value
Fever	54	73	0.061
Coughing	20	63	0.001
Pharyngeal discomfort	3	15	0.058
Diarrhea	1	19	0.002

### Novel coronavirus load comparison

No significant difference was observed in the median CT value of the novel Coronavirus ORF1ab (Table 2) in children [19.80 (5.99–36.58)], and adults [21.01 (7.05–41.00)] (*P* = 0.191). Similarly, the median CT value of the novel Coronavirus nucleocapsid protein N in children was 19.33 (5.66–41.00), and 20.48 (5.80–41.00) in adults (*P* = 0.5440).

**Table 2:** Comparison of laboratory indexes between children and adults

Indicators	Children (80)	Adults (132)	*P value
WBC (10^9^/L)	4.96 (2.14–22.59)	5.47 (1.17-11.00)	0.5562
Decreased WBC (N)	29/80	40/131	0.4500
Lymphocytes (10^9^/L)	1.98 (0.25–4.25)	1.20 (0.29–4.27)	< 0.0001
Decreased lymphocytes (N)	24/80	57/131	0.0584
Interleukin-6 (0–7 pg/mL)	5.87 (1.50–61.40)	15.15 (1.79–166.30)	< 0.0001
Elevated interleukin-6 (n)	32/78	93/131	< 0.0001
Albumin (> 40 g/L)	45.64 ± 3.14	43.31 ± 3.26	< 0.0001
Decreased albumin (N)	1/79	14/113	0.0048
Total bilirubin (5.1–19.0 µmol/L)	4.7 (1.2–12.1)	7.1 (2.2–17.7)	< 0.0001
Elevated total bilirubin (N)	0/79	0/113	> 0.9999
ALT (< 40 U/L)	11.5 (4.6–478.0)	27.2 (4.0–275.0)	< 0.0001
Elevated ALT (N)	4/79	42/113	< 0.0001
AST (< 40 U/L)	25.3 (12.0–228.0)	27.3 (8.8–221.0)	0.3576
Elevated AST (N)	6/79	36/112	< 0.0001
Creatinine (µmol/L)	42.0 (28.0–73.0)	57.0 (32.0–94.0)	< 0.0001
Elevated creatinine (n)	2/79	1/111	0.5714
Proteinuria (N)	22/75	45/112	0.1615
Viral-load (ORF1ab, CT)	19.80 (5.99–36.58)	21.01 (7.05–41.00)	0.1910
Viral-load (N, CT)	19.33 (5.66–41.00)	20.48 (5.80–41.00)	0.5440

Lymphocytes, normal range: 2–6 years old, 1.8–6.3 × 10^9^/L; 6–13 years old, 1.5–4.6 × 10^9^/L; adults, 1.2–3.8 × 10^9^/L; Creatinine (reference intervals, µmol/L): for child, 28 days to < 2 years old, 13–33; 2 to < 6 years old, 19–44; 6 to < 13 years old, 27–66; 13 to < 15 years old, male 37–93, female 33–75, according to *Reference intervals of clinical biochemistry tests commonly used for children* (WS/T 780–2021); for adult, male 20–59 years old, 57–97, 60–79 years old, 57–111; female 20–59 years old, 41–73, 60–79 years old, 41–81, according to *Reference intervals for common clinical biochemistry tests* (WS/T 404.4–2018)

N: The number of people. Because the number of critical cases is small, it is not included in the comparison of laboratory indexes; P value: Comparison of mild and moderate cases in children or adults

*WBC* white blood cells, normal range: 2–6 years old, 4.4–11.9 × 10^9^/L; 6–13 years old, 4.3–11.3 × 10^9^/L; adult, 4.1–10 × 10^9^/L; *ALT* alanine aminotransferase; *AST* aspartate transferase; *CT* cycle threshold (the number of cycles experienced when the fluorescence signal in each reaction tube reaches the set threshold value

*P value: comparison between children and adults

### Blood routine comparison

There was neither a significant difference in the routine blood leukocyte count and the proportion of leukopenia between children and adults (*P* > 0.05; Table 2) nor was a difference observed in children between the lymphocyte count and the proportion of decreased lymphocytes when the mild and moderate groups were compared (*P* > 0.05). For adults, the lymphocyte count was lower in the moderate group than in the mild group, while the proportion of lymphocyte decrease was higher than that of the mild group. The median lymphocyte count was significantly higher (*P* < 0.01) in children [1.98 (0.25–4.25) × 10^9^/L] than in adults [1.20 (0.29–4.27) × 10^9^/L] in adults, but there was no significant difference in the proportion of lymphocyte decrease.

### Interleukin-6 comparison

The median level of IL-6 was 5.87 (1.50–61.40) pg/mL in 78 children 5.29 [(1.50–58.77) pg/mL in the mild group and 6.33 (1.99–61.40) pg/mL in the moderate group]; this difference was not significant. In the adults, the median level of IL-6 was 15.15 (1.79–166.30) pg/mL [5.25 (1.79–36.28) pg/mL in the mild group and 17.44 (2.00–166.30) pg/mL in the moderate group, Additional file 1: Table S1]. The levels in the moderate group were significantly higher than in the mild group (*P* < 0.01). The level of IL-6 in children was also significantly lower than in the adult group (*P* < 0.01). Elevated IL-6 levels were observed in 41.0% (32/78) children (Table 2) with no significant difference between the mild and moderate groups. In the adult group, 93 (71.0%) had an elevated IL-6 (7 mild and 86 moderate) with the elevated rate in the moderate group significantly higher than in the mild group (*P* < 0.01). Elevated IL-6 was significantly lower (*P* < 0.01) in children than in adults (Table 2).

### Comparison of liver function and kidney function

No statistical difference was observed in the levels of albumin when the mild and moderate groups of children were compared (Table 2). A similar finding was observed when comparing the mild and moderate adult groups. However, the mean albumin level in children was significantly higher than in adults (*P* < 0.01). Albumin decrease occurred in only 1/79 (1.3%) children while 14/113 (12.4%) adults had albumin levels below the normal range. The difference in the decrease in albumin levels between children and adults was significant (*P* < 0.01). The total bilirubin in both children and adults were within the normal range [children 4.7 (1.2–12.1) µmol/L and adults 7.1 (2.2–17.7) µmol/L] although the levels in children were significantly lower (*P* < 0.01). The median of alanine aminotransferase (ALT) in children [11.5 (4.6–478.0) U/L], was significantly lower (*P* < 0.01) than in adults [27.2 (4.0–275.0) U/L]. ALT was higher than normal in 4/79 (5.1%) children and in 42/113 (37.2%) adults (*P* < 0.01). The median aspartate transferase (AST) was 25.3 (12.0–228.0) U/L in children and 27.3 (8.8–221.0) U/L in adults, with no significant difference between the two groups (P = 0. 3576). Elevated AST was observed in 6/79 children (7.6%), of whom 2/6 were mild cases and 4/6 were moderate cases of COVID-19. Among the adults, 36/112 patients (32.1%) showed elevated AST, of whom 3/36 had mild and 33/36 had moderate infection. The rate of AST elevation was significantly lower in the children than in adults (P < 0.01). The incidence of liver injury was significantly lower in children than in adults at 8/79 (10.1%) versus 48/113 (42.5%), respectively, (*P* < 0.01).

Serum creatinine levels in the adults were higher than those in the children group [57.0 (32.0–94.0) vs 42.0 (28.0–73.0), *P* < 0.0001]. However, most of them were within the normal range stratified by the age. The frequencies with elevated creatinine levels in both adults and children were rather low (Table 2). Urea nitrogen level was slightly higher in adults than that in children (*P* = 0. 0491). Proteinuria occurred in 22/75 children (29.3%) and 45/112 adults (40.2%; Table 2). However, no significant difference was observed between the two groups (*P* = 0.16).

## Discussion

The novel coronavirus Delta variant was the pathogen of COVID-19 that broke out in Putian, Fujian province in September 2021. Compared with the early epidemic strain, the Delta variant had a higher replication and transmission ability [6]. In this study, there was no significant difference in SARS-CoV-2 viral load between children and adults infected with the novel coronavirus Delta variant. Angiotensin converting enzyme II is a target cell surface receptor for SARS-CoV-2 binding. Although the distribution of ACE2 and immune system characteristics in children are different from those in adults, they still have the same susceptibility to the novel coronavirus Delta variant.

The proportion of mild infections in children (50%) was higher than in adults (17.9%), and so were the proportions of cough and diarrhea symptoms. Children’s clinical symptoms and classification were mild, the possible reasons being as follows: [1] ACE2 is highly expressed in children’s lung tissue, and its expression decreases with age [7], which may cause children’s illness to be mild. [2] The pathogenesis and progress of COVID-19 are not only related to the direct damage of SARS-CoV-2, but also the immune damage caused by the excessive response of the immune system. Children’s immune systems are not yet mature, and it is possible that the activation of immune cells and the release of cytokines are reduced, so the tissue damage is not as serious as that of adults. [3] From the statistical results, it can be seen that there were more comorbidities in adults, than in children, which may also affect the severity of COVID-19.

After SARS-CoV-2 invades the human body, lymphocytes can be consumed and destroyed, resulting in a decrease in peripheral blood lymphocytes. It has been reported that 82.1% of COVID-19 patients have decreased lymphocytes, and 95.5% of them are patients with severe illness [8]. Multivariate logistic regression analysis showed that the lymphocyte count was one of the independent predictors of severe COVID-19. The counts of T cells, CD4^+^ T cells and B cells in patients with severe illness were significantly lower than those in patients with mild illness [9].This study found that the lymphocyte count of children was higher than adults, which is consistent with the clinical classification of children patients. In addition, the physiological increase of lymphocytes in childhood is also one of the reasons for the lymphocyte count in children being higher than in adults.

The cytokine storm induced by the excessive immune response of the host is one of the main pathogenesis mechanisms of COVID-19 [10]. Interleukin-6 (IL-6) is a cytokine of the chemokine family, produced by pulmonary interstitial cells and almost all immune system cells. Overexpression of IL-6 plays a key role in the activation and development of the cytokine storm [11]. Studies have shown that the level of IL-6 in patients with severe COVID-19 was significantly higher than in patients with mild COVID-19 [12, 13]. A meta-analysis involving nine COVID-19 studies showed that the increase of IL-6 was positively correlated with the severity of COVID-19 [14]. The results showed that the level and elevation rate of IL-6 in children were lower than in adults suggesting that the inflammation in the children was mild.

In our previous meta-analysis, we found that there were great differences in liver function indices between patients who died owing to COVID-19 and those who survived [15]. This study found that liver injury occurred in both children and adults. The ACE2, binding receptor of SARS-CoV-2, is highly expressed in bile duct epithelial cells. Thus, SARS-CoV-2 can directly infect bile duct epithelial cells and further cause liver injury. After the body infection with SARS-CoV-2, the release of numerous proinflammatory cytokines and free radicals caused by excessive immune response, can also cause nonspecific inflammation of the liver, and increase ALT, AST and even bilirubin [16]. Possible reasons for the lower frequency of liver injury in children include:

1. The children’s immune systems are not perfect, so the nonspecific inflammation of liver is mild.
2. Some adults use all-human monoclonal neutralizing antibodies, thymosin, and other drugs, to treat their comorbidities, whereas children do not use these drugs. Therefore, drug-induced liver injury in adults, considering these factors, cannot be completely ruled out.
3. According to the national epidemiological survey, the prevalence rates of HBsAg among people aged 1–4, 5–14, and 15–29 years are 0.32%, 0.94% and 4.38%, respectively [17]. Therefore, children may have a lower HBsAg positive rate than adults, which may also cause the difference of abnormal liver function between the two groups.

SARS-CoV-2 binding receptors are not only distributed in the respiratory tract and bile duct epithelium, but are also highly expressed in the kidney, mainly distributed in proximal tubules, afferent arterioles, collecting ducts and thick ascending branches of the Helen duct [18]. SARS-CoV-2 can infect the kidneys by binding with ACE2 or other receptors, which directly leads to kidney injury [19]. After virus infection in the kidney, ACE2 expression can be down-regulated [20], which inhibits the degradation of angiotensin II, which further leads to renal fibrosis [21].

In addition, immune disorders and cytokine storms induced by SARS-CoV-2 can also lead to kidney injury [22]. This study found that proteinuria could occur in both children and adults. The proportion of positive urinary protein is much higher than that of elevated serum creatinine. It is suggested that children with COVID-19 are as prone to kidney injury as adults, and renal tubules are the main site of renal injury. The urinary protein in the children was mainly a little ~ 1+, and proteinuria disappeared quickly with the improvement of the disease, suggesting that kidney injury was mild and recovered easily. The level of serum creatinine in adults was higher. Should be noted, the adults tend to have higher serum creatinine levels than those in children in physiological condition. Therefore, it should be cautious that whether the higher serum creatinine level in the adults group can be attributed to COVID-19. In addition, exogenous creatinine intake, and age also influence serum creatinine levels.

To sum up, differences exist among children and adults infected with the Delta variant COVID-19 in clinical manifestations, clinical classification, inflammatory indices, and biochemical indices. The children’s conditions were relatively mild. In conclusion, compared with the adults with delta variant COVID-19, children tend to have lighter clinical manifestations, higher lymphocyte count, lower IL-6 level, less frequent liver injury, and milder clinical typing. Practitioner should pay attention to the differences in the clinical manifestations between the adults and children. Understanding these differences is helpful to develop tailored treatment plans. The highlights of this study are as follows: (1) the number of children with Delta variant COVID-19 was large, 80 cases; and (2) the clinical symptoms, blood routine, IL-6, liver function, kidney function, urine protein, and viral load of children and adults with the Delta variant of COVID-19 were comprehensively compared. Few articles have compared the clinical characteristics of the two groups in such detail.

Limitations include the following (1) this paper is a retrospective study rather than an analysis and (2) the viral load of SARS-CoV-2 is indicated by a cycle threshold, which is not accurate enough.

## Supplementary Information

**Additional file 1: Table S1.** Comparison of laboratoryindexes between children and adults stratified by clinical classification. 

## Data Availability

The data that support the findings of this study are available in the manuscript and the Additional file 1: Table S1. Data are, however, available from the authors upon reasonable request and with permission of Prof. Qin Li, Email: liqing05912006@163.com.
