# Supplementary material for: Different clinical features of children and adults in regional outbreak of Delta COVID-19
Source: BMC Infect Dis. 2022 Sep 8;22:728. doi: 10.1186/s12879-022-07707-6 (PMC9454403; doi:10.1186/s12879-022-07707-6)
Supplement: Supplementary file 1 — Additional file 1: Table S1. Comparison of laboratoryindexes between children and adults stratified by clinical classification. [file 12879_2022_7707_MOESM1_ESM.docx]

Additional Table S1 Comparison of laboratory indexes between children and adults stratified by clinical classification

| Indicators | Children (80) | | | Adults (132) | | |
| --- | --- | --- | --- | --- | --- | --- |
|  | Mild cases | Moderate cases | P value | Mild cases | Moderate cases | P value |
| Male/Female | 21/19 | 24/16 | 0.6525 | 11/13 | 34/74 | 0.1800 |
| WBC (10^9^/L) | 4.91 (2.14-22.59) | 4.99 (2.48-11.63) | 0.5608 | 6.06 (3.24-11.00) | 5.35 (1.17-10.20) | 0.0442 |
| Decreased WBC (N) | 15/40 | 14/40 | > 0.9999 | 5/23 | 35/108 | 0.4550 |
| Lymphocytes (10^9^/L) | 1.96 (0.25-3.65) | 1.99 (0.40-4.25) | 0.5608 | 1.85 (0.40-4.27) | 1.15 (0.29-2.89) | < 0.0001 |
| Decreased lymphocytes (N) | 12/40 | 12/40 | > 0.9999 | 3/23 | 54/108 | 0.0010 |
| Interleukin-6 (0-7 pg/ml) | 5.29 (1.50-58.77) | 6.33 (1.99-61.40) | 0.1211 | 5.25 (1.79-36.28) | 17.44 (2.00-166.30) | < 0.0001 |
| Elevated Interleukin-6 (n) | 14/40 | 18/38 | 0.3575 | 7/24 | 86/107 | < 0.0001 |
| Albumin (> 40 g/L) | 45.65 ± 2.91 | 45.64 ± 3.39 | 0.9903 | 44.0 ± 3.52 | 43.16 ± 3.20 | 0.3734 |
| Decreased Albumin (N) | 0/39 | 1/40 | > 0.9999 | 2/21 | 12/92 | > 0.9999 |
| Total Bilirubin (5.1-19.0 μmol/L) | 5.0 (1.2-12.1) | 4.5 (2.2-12.1) | 0.6802 | 7.8 (2.2-16.3) | 7.1 (2.7-17.7) | 0.2600 |
| Elevated Total Bilirubin (N) | 0/39 | 0/40 | > 0.9999 | 0/21 | 0/92 | > 0.9999 |
| ALT (< 40 U/L) | 11.5 (4.6-478.0) | 11.5 (5.8-49.0) | 0.9165 | 21.2 (8.6-127.0) | 28.3 (4.0-275.0) | 0.6065 |
| Elevated ALT (N) | 3/39 | 1/40 | 0.3589 | 7/21 | 35/92 | 0.8047 |
| AST (< 40 U/L) | 25.6 (16.4-228.0) | 25.3 (12.0-57.0) | 0.8511 | 25.7 (13.1-72.4) | 27.6 (8.8-221.0) | 0.2087 |
| Elevated AST (N) | 2/39 | 4/40 | 0.6752 | 3/21 | 33/91 | 0.0694 |
| Creatinine (μmol/L) | 42.0 (28.0-57.0) | 42.0 (28.0-73.0) | 0.2473 | 65.0 (43.8-84.0) | 56.5 (32.0-94.0) | 0.4899 |
| Elevated Creatinine (n) | 0/39 | 2/40 | 0.4937 | 0/21 | 1/90 | > 0.9999 |
| Proteinuria (N) | 8/38 | 14/37 | 0.1332 | 10/21 | 35/91 | 0.4674 |
| Viral-load (ORF1ab, CT) | 17.9 (6.0-36.6) | 20.8 (8.0-31.9) | 0.1294 | 23.0 (7.3-28.9) | 20.5 (7.1-41.0) | 0.2264 |
| Viral-load (N, CT) | 18.1 (5.7-41.0) | 20.0 (7.7-41.0) | 0.1462 | 21.7 (6.9-41.0) | 20.1 (5.8-41.0) | 0.1909 |

Notes:

WBC = white blood cells, normal range: 2 ~ 6 years old, 4.4 ~ 11.9 × 10^9^/L; 6 ~ 13 years old, 4.3 ~ 11.3 × 10^9^/L; adult, 4.1 ~ 10 × 10^9^/L;

Lymphocytes, normal range: 2 ~ 6 years old, 1.8~ 6.3 × 10^9^/L; 6~ 13 years old, 1.5~ 4.6 × 10^9^/L; adults, 1.2-3.8 × 10^9^/L;

ALT = Alanine aminotransferase;

AST = Aspartate transferase;

Creatinine (reference intervals, μmol/L): for child, 28 days ~ < 2 years old, 13 ~ 33; 2 years ~ < 6 years old, 19 ~ 44; 6 years ~ < 13 years old, 27 ~ 66; 13 years ~ < 15 years old, male 37~93, female 33~75, according to *Reference intervals of clinical biochemistry tests commonly used for children* (WS/T 780—2021); for adult, male 20 years ~ 59 years old, 57 ~ 97, 60 years ~ 79 years old, 57 ~ 111; female 20 years ~ 59 years old, 41 ~ 73, 60 years ~ 79 years old, 41~81, according to *Reference intervals for common clinical biochemistry tests* (WS/T 404.4—2018);

CT = Cycle threshold (the number of cycles experienced when the fluorescence signal in each reaction tube reaches the set threshold value;

P value: Comparison of mild and moderate cases in children or adults;

^*^P Value: Comparison between children and adults;

N: The number of people. Because the number of critical cases is small, it is not included in the comparison of laboratory indexes.

[2] World Health Organization. Tracking SARS-CoV-2 variants. 2022 [cited 2022-01-04]; Available from: https://www.who.int/en/activities/tracking-SARS-CoV-2-variants/

[3] Di Giacomo S, Mercatelli D, Rakhimov A, Giorgi FM. Pre-critical report on severe acute response syndrome coronvirus 2 (SARS-CoV-2) Spike mutation T478K. J Med Virol 2021; 93 (9): 5638-5643.

[4] Chen J, Jiang Q, Xia X, Liu K, Yu Z, Tao W, et al. Individual variation of the SARS-CoV-2 receptor ACE2 gene expression and regulation. Aging Cell 2020; 19 (7).

[5] Yang L, He Q.Research progress on the role of angiotensin converting enzyme 2 in the pathogenesis and treatment of novel coronavirus pneumonia (COVID-19). Chinese Journal of Pharmacology and Toxicology 2020; 34 (8): 575-583.

[6] Deng Quanmin, Liang Ping, Liu Hanmin. Epidemiological characteristics and mechanism analysis of novel coronavirus pneumonia in children. Chinese Journal of Contemporary Pediatrics 2021; 23 (4): 420-424.

[7] Guan WJ, Ni ZY, Hu Y, Liang WH, Ou CQ, He JX, et al. Clinical Characters of Coronavirus Disease 2019 in China. N Engl J Med 2020; 382 (18): 1708-1720.

[8] Wu D, Wu X, Huang J, Rao Q, Zhang Q, Zhang W. Lymphocyte subset alterations with disease severity, imaging manifestation, and delayed hospitalization in COVID-19 patients.BMC Infect Dis 2021; 21 (1): 631.

[9] Pinzon RT, Wijaya VO, Buana RB. Interleukin-6 (IL-6) thieves as therapeutic agents for coronvirus disease 2019 (COVID-19): A systematic review and meta-analysis. J Infect Public Health 2021; 14 (8): 1001-1009.

[10] Magro G. SARS-CoV-2 and COVID-19: Is interleukin-6 (IL-6) the'culprit lesion 'of ARDS onset? What is there besides Tocilizumab? SGP130FC. Cytokine X 2020; 2 (2): 10,0029.

Fang Yicheng, Zhang Huangqi, Xie Jicheng, Lin Minjie, Lin Shuangxiang, Yang Xiaochen, et al. Comparison and clinical significance of lymphocyte ratio, IL-6 and IL-10 levels in novel coronavirus pneumonia with different clinical types. Zhejiang Medicine 2021; 43 (7): 763-765.

[12] Aziz M, Fatima R, Assaly R. Elevated interleukin-6 and severe COVID-19: A meta-analysis. J Med Virol 2020; 92 (11): 2283-2285.

[13] Xing QQ, Dong X, Ren YD, Chen WM, Zeng DY, Cai YY, et al. Liver Chemicals in Patients with COVID-19 Who Discharged Live or Died: A Meta-analysis. Hepatol Commun 2021; 5 (1): 12-23.

[14] Li Lu, Li Shuang, Chen Yu.Mechanism of liver injury in novel coronavirus pneumonia. Journal of Clinical Hepatobiliary Diseases 2021; 37 (1): 209-211.

[15] Infectious Diseases Branch of Chinese Medical Association, Hepatology Branch of Chinese Medical Association. Guidelines for Prevention and Treatment of Chronic Hepatitis B (2019 Edition). Journal of Clinical Hepatobiliary Diseases 2019; 35 (12): 2648.

[16] Santos RA, Ferreira AJ, Verano-Braga T, Bader M. Angiotensin-converting enzyme 2, angiotensin-(1-7) and Mas: new players of the renin-angiotensin system. J Endocrinol 2013; 216 (2): R1-R17.

[17] Qian JY, Wang B, Liu BC. Acute Kidney Injury in the 2019 Novel Coronavirus Disease. Kidney Dis (Basel) 2020; 323 (1-6.

[18] Yan Yuehua, Yang Chunhui, Kang Xiaowen, Yue Rui, Wang Xiexi, Chen Liting, et al. Study on the mechanism of kidney injury in novel coronavirus pneumonia. Chinese Journal of Integrated Traditional Chinese and Western Medicine Nephrology 2021; 22 (5): 438-439.

[19] Qu Zhuan, Wang Huiming. Study on the mechanism of kidney injury in novel coronavirus pneumonia (COVID-19). Journal of Difficult Diseases 2021; 20 (5): 527-531.
